# Quercetin ameliorates diabetic encephalopathy through SIRT1/ER stress pathway in db/db mice

**DOI:** 10.18632/aging.103059

**Published:** 2020-04-20

**Authors:** Tian Hu, Jing-Jing Shi, Jiansong Fang, Qi Wang, Yun-Bo Chen, Shi-Jie Zhang

**Affiliations:** 1 Science and Technology Innovation Center, Guangzhou University of Chinese Medicine, Guangzhou, China; 2 Institute of Clinical Pharmacology, Guangzhou University of Chinese Medicine, Guangzhou, China; 3 Department of Neurology, The Second Affiliated Hospital of Guangzhou University of Chinese Medicine, Guangzhou, China

**Keywords:** quercetin, diabetic encephalopathy, SIRT1, ER stress

## Abstract

Studies have shown that diabetes is an important risk factor for cognitive dysfunction, also called diabetic encephalopathy (DE). Quercetin has been reported to be effective in improving cognitive dysfunction in DE. But its detailed mechanism is still ambiguous. In this study, we used db/db mice to investigate whether quercetin could activate SIRT1 and inhibit ER pathways to improve DE. Behavioral tests (Morris water maze and new objects) showed that quercetin (70 mg/kg) can effectively improve the learning and memory ability in db/db mice. OGTT and ITT tests indicated that quercetin could alleviate impaired glucose tolerance and insulin resistance in db/db mice. Western blot analysis and Nissl staining showed that quercetin can improve the expression of nerve and synapse-associated proteins (PSD93, PSD95, NGF and BDNF) and inhibit neurodegeneration. Meanwhile, quercetin up-regulates SIRT1 protein expression and inhibits the expression of ER signaling pathway-related proteins (PERK, IRE-1α, ATF6, eIF2α, BIP and PDI). In addition, oxidative stress levels were significantly reduced after quercetin treatment. In conclusion, current experimental results indicated that SIRT1/ER stress is a promising mechanism involved in quercetin-treated diabetic encephalopathy.

## INTRODUCTION

Diabetic encephalopathy (DE) is one of the major complications of diabetes, which Is characterized by a series of neurophysiological and pathological structural abnormalities caused by hyperglycemia [1, 2]. In the central nervous system, neuronal function in type 1 and type 2 diabetes models are reduced, and neuron numbers are significantly reduced [3, 4]. Brain insulin signaling system changes [5] cerebrovascular abnormalities [6], oxidative stress [7], and increased advanced glycation end products [8] are considered to be potential causes of DE.

Endoplasmic reticulum (ER) is an important organelle for the synthesis of proteins, glycogens, steroids and lipids to maintain cell homeostasis in eukaryotic cells [9]. Protein disulfide isomerase (PDI) and an immunoglobulin binding protein (BIP) assist in the correct folding of proteins on the ER. Calcium ion imbalance, oxidative stress, and many other factors lead to unfolded or misfolded proteins causing ER stress, also called unfolded protein response (UPR) [10, 11]. It has been reported that ER stress accelerates the death of diabetic retinopathy and nerve cells [12, 13]. Abnormal glucose levels and insulin signaling trigger ER stress, which induces neuronal apoptosis [14].

Quercetin, a common flavonoid, is widely distributed in daily intake of fruits and vegetables [15]. As a potent antioxidant, quercetin is effective in scavenging free radicals, inhibiting xanthine oxidase activity and lipid peroxidation [16, 17]. Quercetin has been reported to have a number of beneficial effects, including anti-cancer [15], anti-inflammatory [18, 19], anti-oxidant [20], hypoglycemic properties [21]. In the development of neurodegenerative diseases, quercetin can improve behavioral dysfunction and memory impairment [17, 22, 23]. Studies have found that quercetin is a potent activator of SIRT1 [24–26]. In an aging rat model, quercetin up-regulates SIRT1, promotes monoamine synthesis in rats, and then improves its cognitive function [24]. Meanwhile, quercetin activates SIRT1 and promote glycometabolism in diabetic rats [27]. Although studies have reported that quercetin promotes improved learning and memory in diabetic rats [28], the mechanism of action of quercetin on DE has not been clearly reported. Therefore, SIRT1 pathway has very important research value.

SIRT1, a deacetylase, is a member of the sirtuin family, and which is involved in the development of cell differentiation, proliferation, senescence and apoptosis [29]. Cardiovascular disease studies have found that inhibition or deficiency of SIRT1 in SIRT1 knockout mice increases ER stress-induced heart damage [30]. Methyl derivative deficiency (MDD) activates the ER stress pathway and reduces SIRT1 expression in rat model of colitis. Interestingly, the activation of SIRT1 protein was accompanied by a decrease in UPR after the addition of SIRT1 activator (SRT1720) [31]. In our previous study, SIRT1 activation could alleviate ER stress and protect cognitive function in diabetic mice [10]. However, whether SIRT1/ER stress pathway participated in quercetin on DE is still unknown.

In this study, we focused on the mechanism of action of quercetin in improve DE. We used an animal model of type 2 diabetes (db/db mice) [10] to investigate whether quercetin improves cognitive dysfunction through the SIRT1/ER stress pathway. We found that quercetin activated SIRT1 and regulated the ER stress pathway might be an effective mechanism in db/db mice.

## RESULTS

### Quercetin improves learning and memory impairment in db/db mice

**Figure 1:**
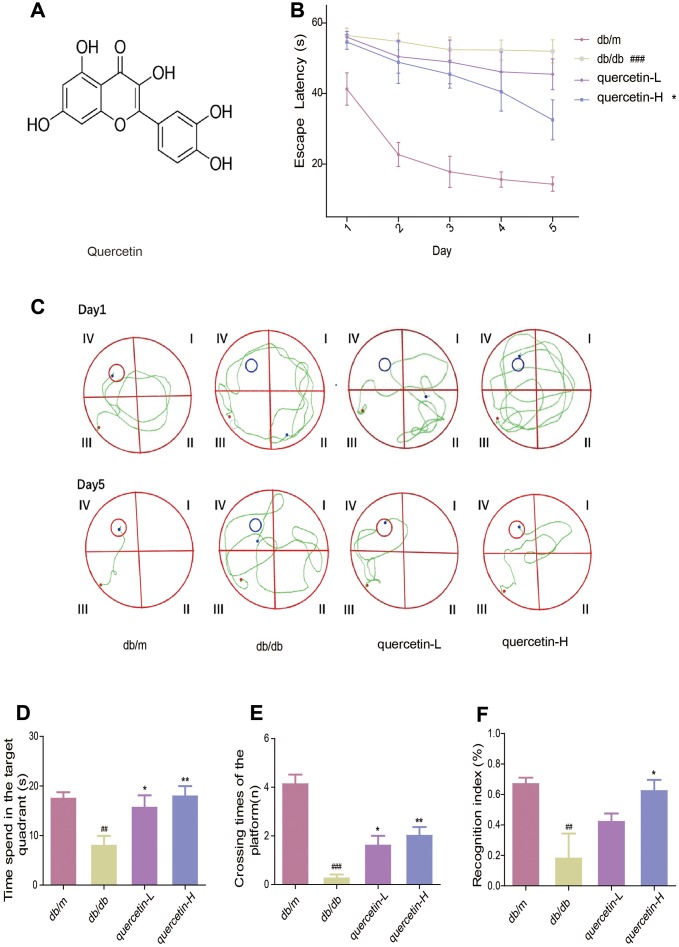
**Quercetin improves learning and memory impairment in db/db mice.** (**A**) The chemical structure of Quercetin. (**B**) Escape latency of the five day in MWM. (**C**) Swimming paths of the db/db mice on the first and fifth day in MWM. (**D**) Time spent in the target quadrant in in MWM. (**E**) Crossing times of the target platform in MWM. (**F**) Novel object preference index in NOR. Quercetin-L: 35mg/kg/d; Quercetin-H: 70mg/kg/d. Data represent mean ± SEM (n = 10 per group). #*p* < 0.05, ##*p* < 0.01, ###*p* < 0.001vs. db/m; * *p* < 0.05, ** *p* < 0.01, *** *p* < 0.001 vs. db/db.

To measure the memory and learning abilities of db/db mice, we performed Morris water maze and new object recognition test. In the Morris water maze test, the time it takes for the mouse to find the central platform is decreasing (Figure 1B). Compared with the db/m group, the db/db group significantly increased the time to find the platform. After treatment with quercetin, the time to find the platform was significantly shorter, especially in the high dose group (Figure 1B). After five days’s training, the swimming path of db/db was more disordered than db/m, while the quercetin-treatment group was significantly improved (Figure 1C). In addition, after the platform was removed, the numbers of exploration of the db/db group in the platform area and the time spend in the target quadrant were significantly reduced. Quercetin-treatment group reversed the phenomena (Figure 1C, 1D).

In the new object recognition test, the TNI level of the db/db group was significantly lower than db/m (Figure 1E). After treatment with quercetin, the mice exhibited better performance than the db/db group. These results indicated that quercetin could significantly improve cognitive deficits in db/db mice.

### Quercetin alleviates impaired glucose tolerance and insulin resistance in db/db mice

**Figure 2:**
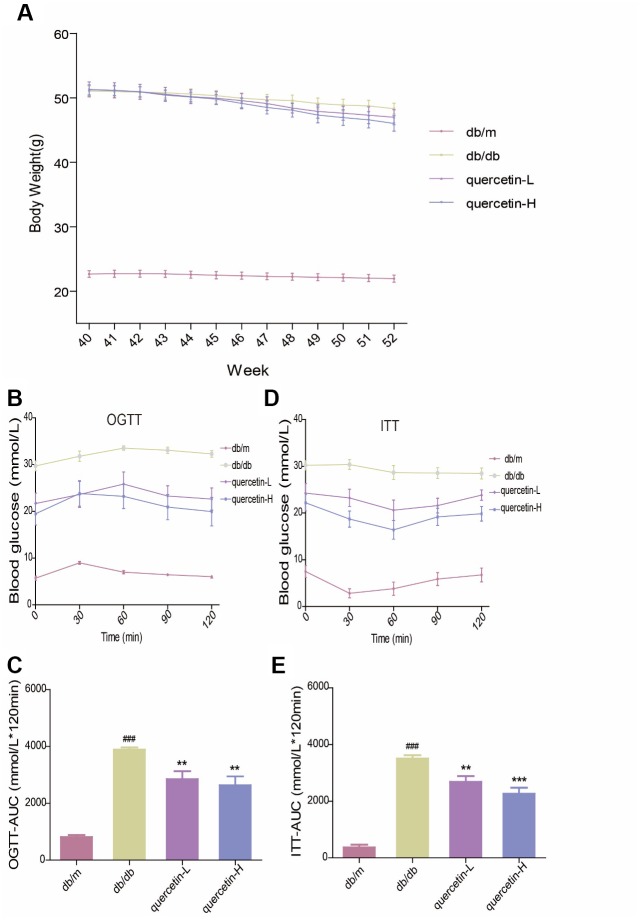
**Quercetin alleviates impaired glucose tolerance and insulin resistance in db/db mice.** (**A**) Body Weight. (**B**) OGTT. (**C**) OGTT-AUC. (**D**) ITT. (**E**) ITT-AUC. Quercetin-L: 35mg/kg/d; Quercetin-H: 70mg/kg/d. Data represent mean ± SEM (n = 10 per group). #*p* < 0.05, ##*p* < 0.01, ###*p* < 0.001vs. db/m; * *p* < 0.05, ** *p* < 0.01, *** *p* < 0.001 vs. db/db.

In the OGTT test, the blood glucose level and the area under the curve at each test time point of the db/db mice were significantly higher than those in the db/m group. However, after 12 weeks of quercetin treatment, the blood glucose level was significantly lower, especially in the high-dose group (Figure 2B, 2C). In the ITT test, insulin sensitivity in db/db mice was significantly lower than in the db/m group (Figure 2D, 2E). After 12 weeks of quercetin treatment, insulin sensitivity and area under the corresponding curve were relatively improved. These results demonstrated that quercetin could reduce fasting blood glucose and improves glucose tolerance and insulin resistance.

### Quercetin decreases oxidative stress in db/db mice

**Figure 3:**
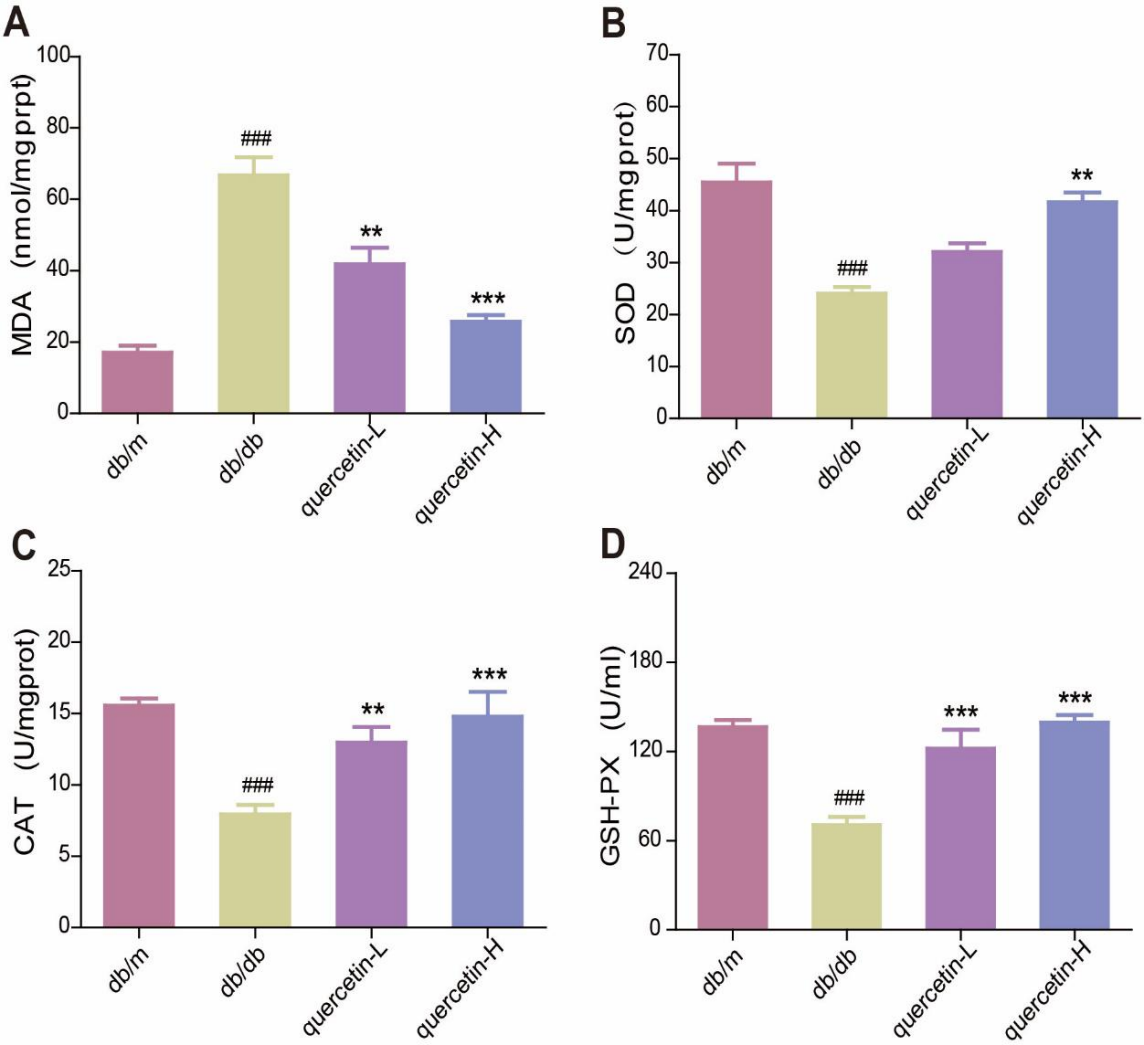
**Quercetin decreases oxidative stress in db/db mice.** (**A**) MDA. (**B**) SOD. (**C**) CAT. (**D**) GSH-PX. Quercetin-L: 35mg/kg/d; Quercetin-H: 70mg/kg/d. Data represent mean ± SEM (n = 10 per group). #*p* < 0.05, ##*p* < 0.01, ###*p* < 0.001vs. db/m; * *p* < 0.05, ** *p* < 0.01, *** *p* < 0.001 vs. db/db.

In the brain of db/db mice, the level of endogenous lipid peroxide MDA increased, and the activity of SOD, CAT and GSH-PX were significantly reduced (Figure 3A–3D). Quercetin significantly relieved the oxidative stress when compared with the db/db group. These results showed that quercetin could remarkably decrease the level of oxidative stress in db/db mice.

### Quercetin ameliorates neurodegeneration in db/db mice

**Figure 4:**
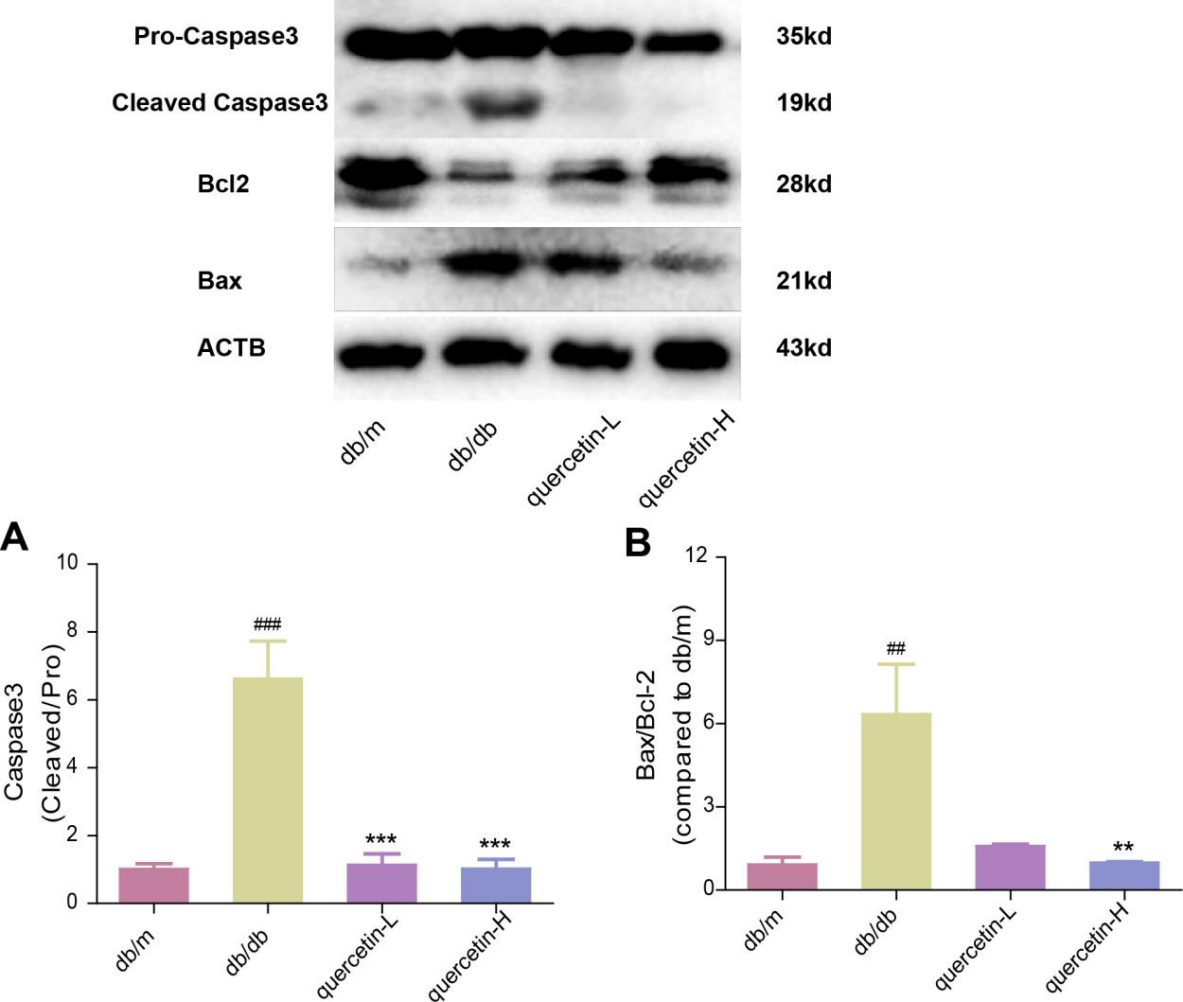
**Quercetin protects against neuronal apoptosis in the brain of db/db mice.** Western blot analysis: (**A**) Caspase3; (**B**) Bax/Bcl2. Quercetin-L: 35mg/kg/d; Quercetin-H: 70mg/kg/d. Data represent mean ± SEM (n = 10 per group). #*p* < 0.05, ##*p* < 0.01, ###*p* < 0.001vs. db/m; * *p* < 0.05, ** *p* < 0.01, *** *p* < 0.001 vs. db/db.

**Figure 5:**
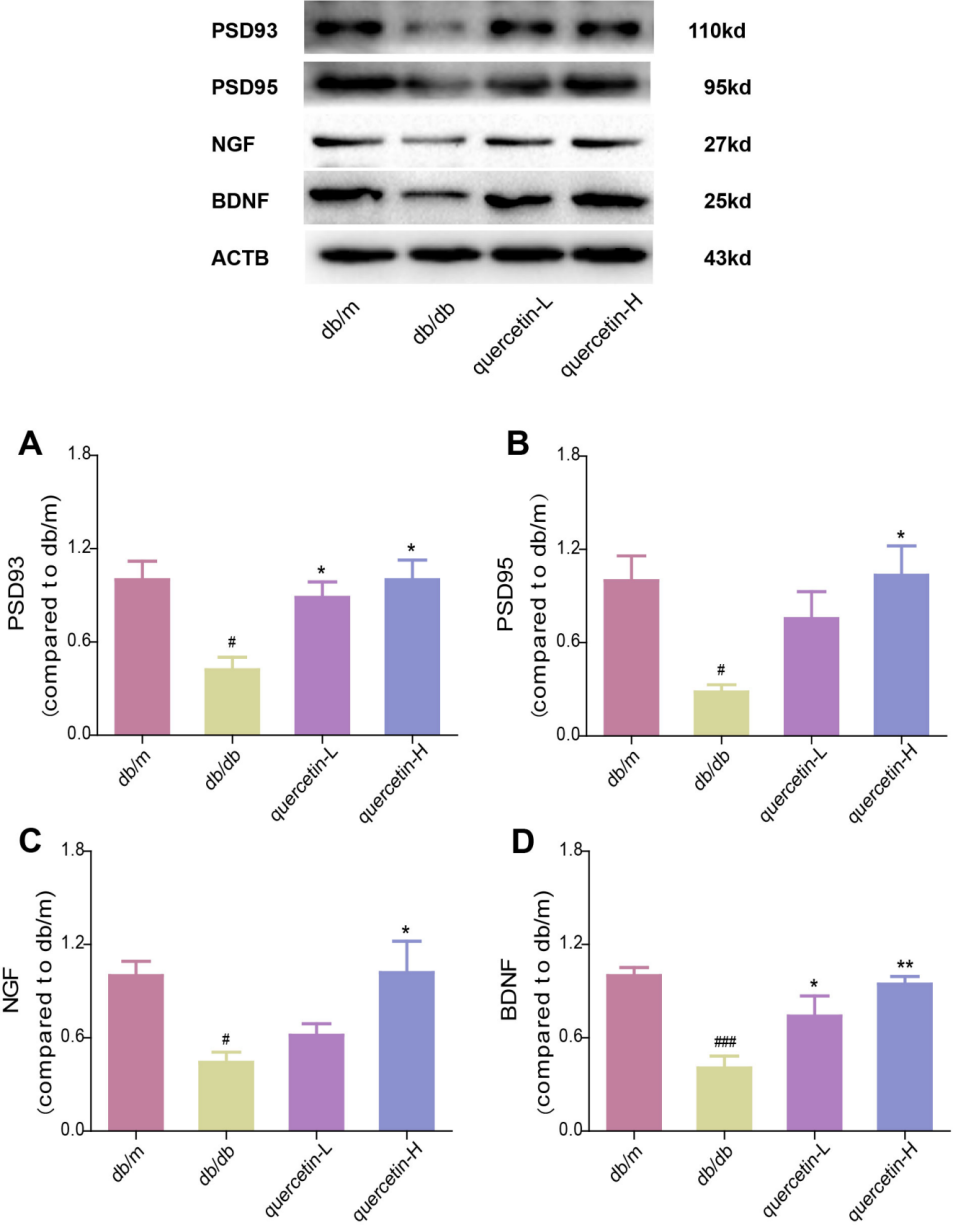
**Quercetin increases neurotrophic factor levels in the brain of db/db mice.** Western blot analysis: (**A**) PSD93; (**B**) PSD95; (**C**) NGF; (**D**) BDNF. Quercetin-L: 35mg/kg/d; Quercetin-H: 70mg/kg/d. Data represent mean ± SEM (n = 10 per group). #*p* < 0.05, ##*p* < 0.01, ###*p* < 0.001vs. db/m; * *p* < 0.05, ** *p* < 0.01, *** *p* < 0.001 vs. db/db.

**Figure 6:**
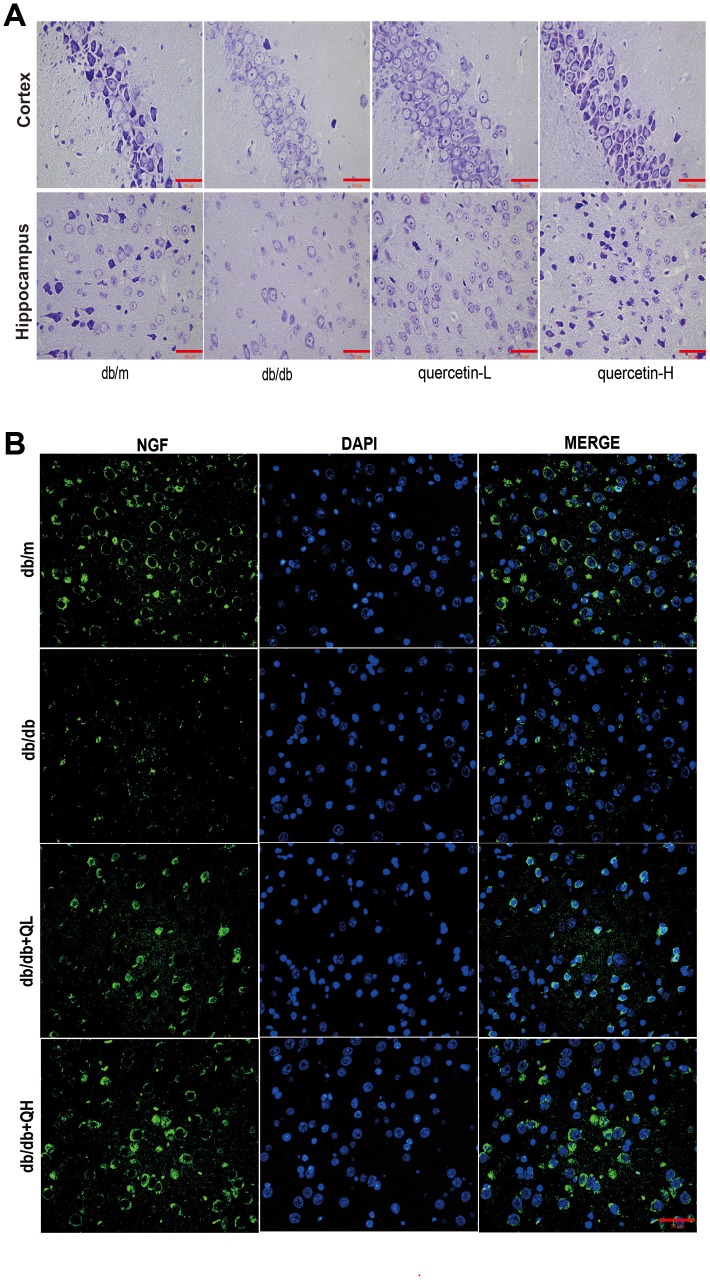
**Quercetin ameliorates neurodegeneration in db/db mice.** (**A**) Nissl’s staining. (**B**) Immunofluorescence of NGF. Scale bar: 100 μm.

In the brain tissue of db/db mice, the expression of proapoptotic protein Bax and cleaved Caspase3 protein increased significantly, and the expression of apoptosis-inhibiting protein Bcl-2 was relatively decreased (Figure 4). After 12 weeks of quercetin treatment, Bcl-2 expression was increased, and the expressions of Bax and cleaved Caspase-3 were sharply reduced. In addition, the expression of neurotrophic factors (BDNF, NGF) and synaptic proteins (PSD93, PSD95) was significantly reduced in db/db mice (Figures 5 and 6B). Quercetin significantly improved the expression of neurotrophic factors and synapse-related proteins. Nissl staining was further verified this change (Figure 6A). In the hippocampal and cortical areas of db/db mice, Nissl body was largely lost and stained weakly. Notably, after quercetin administration, these neurons were found a deeper and denser Nissl body. These results indicated that quercetin could protect against neurodegeneration in db/db mice.

### Quercetin activates SIRT1 and relieves ER stress in db/db mice

**Figure 7:**
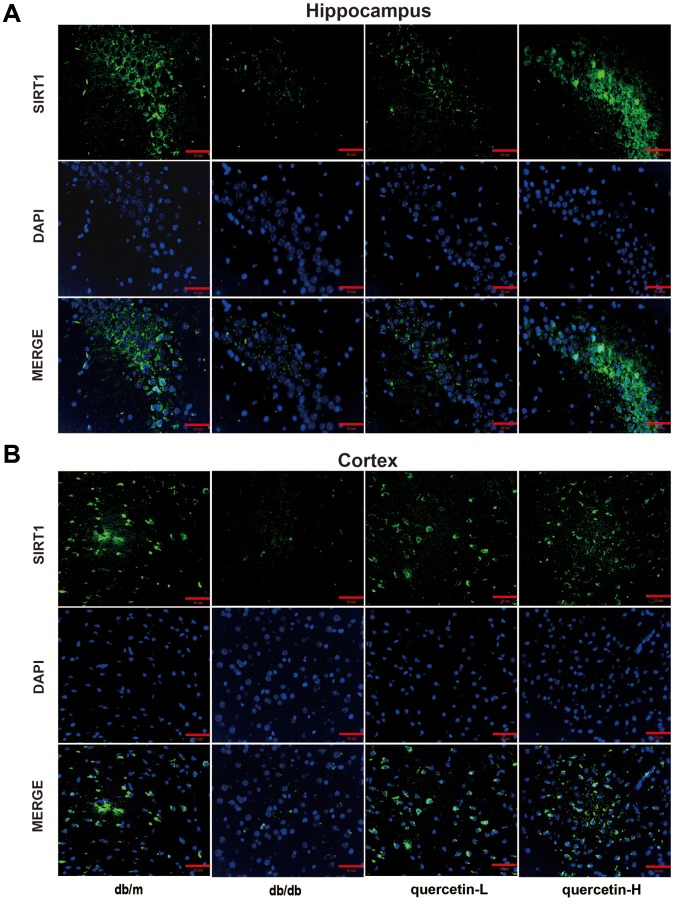
**Quercetin activates SIRT1 in the brain of db/db mice.** (**A**) Immunofluorescence of SIRT1 in hippocampus. (**B**) Immunofluorescence of sirt1 in cortex. Scale bar: 100 μm.

**Figure 8:**
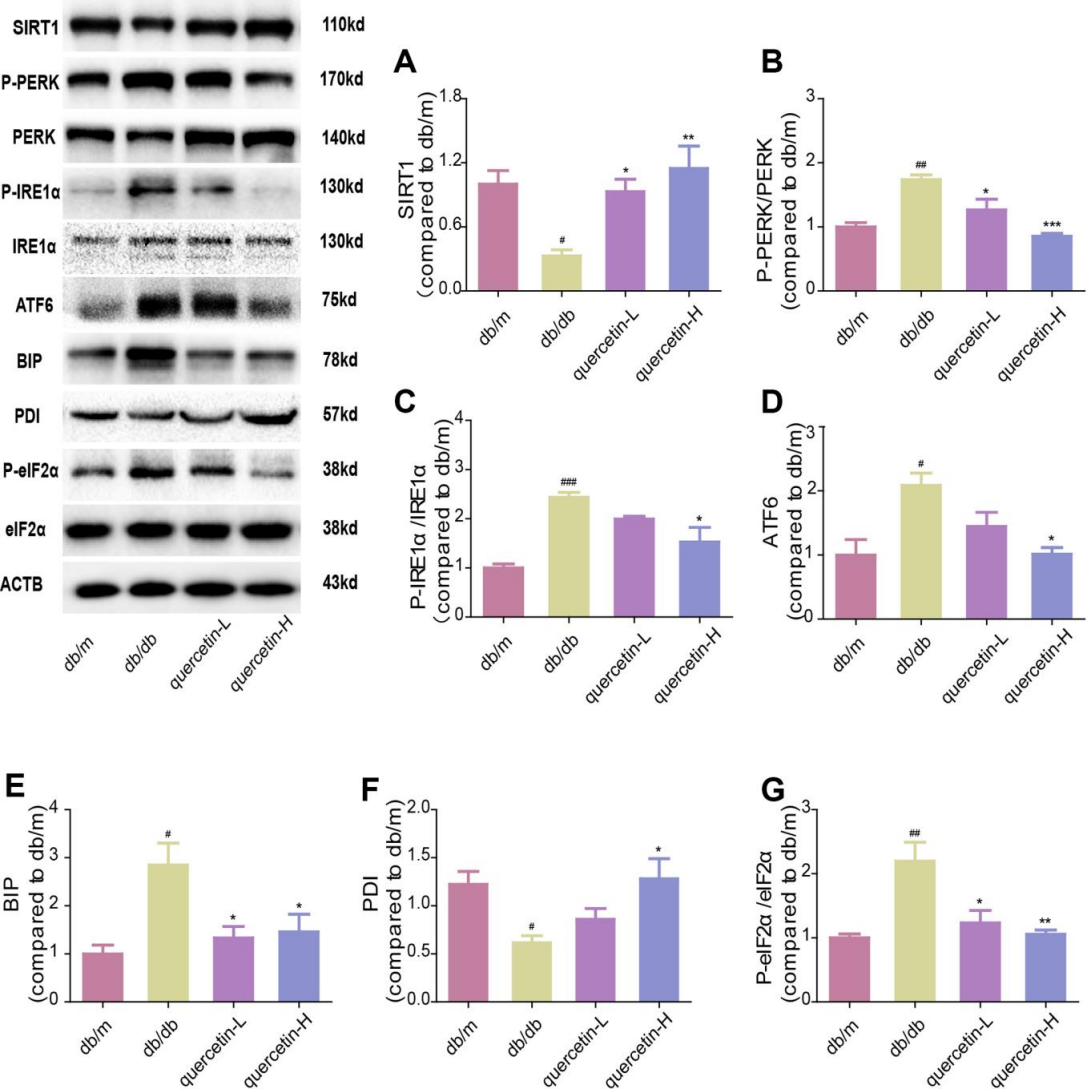
**Quercetin activates SIRT1 and relieves ER stress in db/db mice.** Western blot analysis: (**A**) SIRT1; (**B**) P-PERK/PERK; (**C**) P-IRE1α/IRE1α; (**D**) ATF6; (**E**) BIP; (**F**) PDI; (**G**) P-eIF2α/eIF2α. Quercetin-L: 35mg/kg/d; Quercetin-H: 70mg/kg/d. Data represent mean ± SEM (n = 10 per group). #*p* < 0.05, ##*p* < 0.01, ###*p* < 0.001vs. db/m; * *p* < 0.05, ** *p* < 0.01, *** *p* < 0.001 vs. db/db.

In both immunofluorescence and western blot results, SIRT1 protein expression was lower in db/db group (Figures 7 and 8). Quercetin, especially in the high dose group, is effective in increasing the protein expression of SIRT1. Subsequently, we measured these proteins expression levels of ER stress-related proteins (Figure 8). In the db/db group, the expression of ER stress marker protein (BIP, PDI) and UPR active protein (P-PERK, P-IRE-1α, P-eIF2, ATF6) were higher than those in the db/m group. After quercetin administration, the expression of these proteins was drastically reduced compared to the db/db group. Taken together, these results demonstrated that quercetin could activate SIRT1 and relieve ER stress to protect DE.

## DISCUSSION

In this study, we demonstrated that quercetin could ameliorate DE. After 12 weeks’ treatment, we found that quercetin could relieve learning and memory dysfunction, reduce fasting blood glucose, and increase insulin sensitivity. Meanwhile, quercetin signally inhibited oxidative stress, ameliorated neurodegeneration. Furthermore, quercetin activated SIRT1 and inhibited the expression of ER stress-related proteins, which may be the key neuroprotective mechanism of quercetin.

Diabetes is caused by insulin synthesis or secretion damage, which leads to hyperglycemia [32]. High glucose environment and insulin signal changes in the brain are the key to DE [10]. Hyperglycemia exacerbates oxidative damage and a range of neurochemical and structural abnormalities in the brain [33, 34]. Studies have shown that quercetin is effective in treating STZ-induced diabetes [26]. The learning and memory abilities of diabetic rats can be significantly improved by quercetin [35]. In this experiment, we further investigated the neuroprotective mechanism of quercetin on db/db mice. Behavioral studies confirmed that quercetin could significantly improve learning and memory in db/db mice. In addition, OGTT and ITT results indicated that quercetin attenuated impaired glucose tolerance and insulin resistance in db/db mice. These effect of quercetin on DE is consistent with previous studies [23, 28].

Neuronal apoptosis and oxidative stress are common pathological features in the pathogenesis of neurodegenerative diseases [36, 37]. The impaired learning and memory abilities of T1DM and T2DM are closely related to changes in hippocampal synapses [36, 38, 39]. Changes in glucose and insulin in the diabetes model lead to oxidative stress [37, 40]. Interestingly, oxidative stress is also a key risk drivers of apoptosis in the model of diabetes [41, 42]. In this study, we found the quercetin markedly attenuated oxidative stress in db/db mice. Simultaneously, quercetin could effectively inhibit neuronal apoptosis in db/db mice. Nissl staining results also demonstrated the neuroprotective effects of quercetin.

SIRT1 is an important member of the sirtuins family, which is involved in many aspects such as inflammation, apoptosis, and oxidative stress [43, 44]. The SIRT1 signaling pathway may be a key mechanism for alleviating diabetes-related neurodegenerative diseases [27]. In our previous study, we found that activation of SIRT1 improved learning and memory in db/db mice [10]. Quercetin is a potent activator of SIRT1. Therefore, we reasonably hypothesized that quercetin could attenuate DE by activating SIRT1 pathway. Our results showed that quercetin significantly increased the expression of SIRT1.

Up-regulation of SIRT1 can inhibit three signaling pathways (IRE1α, PERK, ATF6) that activate ER stress [44, 45]. The downstream, PERK and eIF2α, have been shown to be positively correlated with cognitive dysfunction [46]. Our previous study indicated that ER stress is found in the hippocampus of db/db mice [10]. In this study, we examined the ER stress pathway in the brain of db/db mice. Our results indicated that quercetin can significantly activate SIRT1 to inhibit ER stress and thereby protect DE in db/db mice.

In summary, this study demonstrated that quercetin protects DE by SIRT1/ER stress pathway. The SIRT1/ER stress pathway provides a reliable reference for the prevention and control of DE. However, there still needs a deeper exploration. This study might provide the possibility of quercetin for DE treatment.

## MATERIALS AND METHODS

### Chemical reagents

Quercetin (CAS NO: 117-39-5, purity > 95%, Figure 1A) was purchased from Sigma-Aldrich. Kits for detecting SOD and MDA were purchased from Nanjing Jiancheng Bioengineering Institute. The antibodies for PSD93, PSD95, SIRT1, Caspase3, Bax, Bcl2, PERK, P-PERK, eIF2α, P-eIF2α, IRE-1α, P-IRE-1α, BIP, PDI were provided by Cell Signaling Technology (MA, USA). Anti-β-actin, BDNF, NGF, ATF6 were purchased from Abcam, Inc (Cambridge, England).

### Animals and treatment

The 10-week-old female diabetic mice (db/db) and age-matched non-diabetic mice (db/m) were from the Model Animal Research Institute of Nanjing University (Nanjing, China). The mice were in SPF animal room, where is a 12-h light/dark cycle at a relative humidity of 40-60 % and temperature 20-25 °C. Then, animals were allowed free access to food and water. After 30 weeks, fasting blood glucose > 11.1 mmol/L was defined as the success criterion for diabetic model. The mice were randomly divided into four groups: db/m (0.9 % saline, n = 10), db/db (0.9 % saline, n = 10), low dosage of quercetin (35 mg/kg/day, n = 10) high dosage of quercetin (70 mg/kg/day, n = 10). Drugs were administered for 12 weeks by gavage. In addition, our experiments are in strict compliance with the guidelines promulgated and adopted by the NIH.

### Morris water maze test

Morris water maze is a test for evaluating spatial reference memory in mice. The method of operation is based on previous studies [10, 47, 48].

### Novel object recognition (NOR) test

NOR, a method for exploring animal recognition and memory of new objects, is based on the instinct of animals to explore the characteristics of new objects [49]. The computer device recorded the time spent by the mice on three objects, respectively, and evaluated the learning and memory ability of the mice using the recognition index (TNI) = (TN-TF) / (TN + TF) [50, 51] (TN: new object time; TF: old object time).

### Oral glucose tolerance test and insulin tolerance test

In OGTT test, animals were fasted for 16 h, and 2 g/kg glucose solution was orally administered by body weight. Then, glucose levels at five time points (90, 30, 60, 90 and 120 min) were measured with a glucose analyzer (ACCU-CHEK, Roche Diagnostics, Basel, Switzerland). Three days later, we continued the ITT experiment. Four hours after fasting, animals were injected intraperitoneally with insulin (0.5 U/kg, Eli Lilly and Co., IN). Blood glucose levels at five time points (0, 30, 60, 90, and 120 min) were measured and the index of the total glucose shift was calculated AUC [36].

### Measurement of SOD, MDA, CAT and GSH-PX

After ITT experiments, all mice were anesthetized with chloral hydrate (0.04 mL/10g, intraperitoneal injection), then they were sacrificed by cervical dislocation. Then, we took an appropriate amount of brain tissue, placed it in four degrees of normal saline, homogenized, centrifuged, and took the supernatant. The corresponding SOD, MDA, CAT and GSH-PX levels were tested according to the kit instructions.

### Nissl staining

The perfused mouse brain was embedded in paraffin and cut into 5 μm thick sections from the coronal plane. Dewaxing, rehydration, dyeing, dehydration, and transparency were sequentially performed according to the kit instructions. Images were then analyzed using an optical microscope (Life Technologies, Leica).

### Immunohistochemistry

The perfused mouse brain was embedded in paraffin and cut into 5 [mu]m thick sections from the coronal plane. Three paraffin sections were taken from each group for dewaxing and rehydration. Antigen retrieval was performed in an antigen retrieval solution (sodium citrate buffer) by microwave heating. Blocking with 5% normal goat serum in PBS at 37°C for 30 min, and then incubating with primary antibody SIRT1 (1:200; CST) or NGF (1:200; Abcam) overnight at 4 °C. After rewarming for 30 minutes, the secondary antibody was incubated for 1 h at 37 °C.

### Western blot analysis

The brain tissue of the mice was taken at -80 °C. Appropriate amount of brain tissue was lysed, homogenized, and then centrifuged for 15 min (4 °C). The supernatant of the tissue after centrifugation was taken. The corresponding protein concentration was measured using a BCA protein assay kit. The expressions of these target proteins (SIRT1, PERK, P-PERK, eIF2α, P-eIF2α, IRE-1α, P-IRE-1α, ATF6, BIP, PDI, Caspase3, Bax, Bcl2, PSD93, PSD95, BDNF, NGF) were then detected by Western blotting.

### Statistical analysis

Ours experimental values were all presented as mean ± SEM. And statistical analyses were all performed using SPSS 19.0 program (IBM, Endicott, NY). Statistical differences in data between groups were performed with ANOVA, and followed by a post hoc test (Dunnett). *P* < 0.05 was presented as statistically significant.
